# Exploring the Views of Children With Cerebral Palsy, Their Parents and Physiotherapists on Participating in a Feasibility Randomised Controlled Trial Testing an Exergaming Device: A Qualitative Study

**DOI:** 10.1111/hex.70533

**Published:** 2025-12-25

**Authors:** Rachel Rapson, Jos M. Latour, Jonathan Marsden, Bernie Carter

**Affiliations:** ^1^ Peninsula Allied Health Centre University of Plymouth Plymouth UK; ^2^ Children and Family Health Devon Torbay and South Devon NHS Foundation Trust Barnstaple UK; ^3^ school of Nursing and Midwifery, Faculty of Health University of Plymouth Plymouth UK; ^4^ Faculty of Health, Social Care and Medicine Edgehill University Liverpool UK

**Keywords:** cerebral palsy, children, exercise device, exergaming, feasibility, gaming, physiotherapy

## Abstract

**Background:**

This study aimed to understand the experiences and views of children with cerebral palsy, their parents and physiotherapists participating in the ACCEPT feasibility randomised controlled trial, which explored a 10‐week physiotherapy intervention using an interactive gaming training device.

**Design:**

Qualitative methods included semi‐structured interviews, e‐diary and photographs. Nine parent–child dyads and three physiotherapists participated. Children were aged between 7 and 16 years; four had co‐existing additional needs. Interviews were transcribed. Reflexive thematic analysis was used.

**Results:**

Five themes covered the breadth of participants’ experiences: (1) Fitting in therapy; (2) Motivation; (3) New opportunities; (4) Physiotherapists out of their comfort zone; and (5) Altruism and challenges.

Parents spoke of the challenge of finding time to engage their children in therapeutic exercise. Several children talked about trial‐related procedures (e.g. removal of adhesive markers) that they disliked. Physiotherapists, children and parents supported the gaming aspect of the device to improve motivation to exercise.

**Conclusions:**

Overall, the intervention and measures were acceptable to children and parents. Parents were willing to accommodate the device for 10 weeks and children found the gaming aspect both motivating and enjoyable. Physiotherapists required more support to solve technical problems with the device. A full trial evaluating the device requires additional technical support from the supplier and experienced ‘champion’ users. Exercise trainers that encompass gaming may increase motivation and adherence to therapeutic programmes.

**Patient or Public Contribution:**

Children, families and care givers were included in the study design. They helped to produce participant facing documents including the e‐diary and interview topic guides. The study steering committee included several parents and one teenager, and provided governance and oversight of the project.

## 1 Introduction

Cerebral palsy (CP) is a common disability with its onset in childhood, resulting from a non‐progressive lesion to the brain [1]. The prevalence of CP is 2–3 per 1000 live births [2]. Motor impairments are classified using the Gross Motor Function Classification System (GMFCS) [3]. Children with GMFCS levels I–III are able to walk with or without aids while children with GMFCS IV and V use wheelchairs for their mobility. Motor impairments associated with CP are spasticity, weakness and reduced selective movement, which interfere with aspects of balance and walking. Children who have motor difficulties report reduced levels of independence and reduced participation at school [4].

Children with CP commonly undertake physiotherapy programmes aimed to help, improve or maintain their motor skills. In recent years the potential of serious games using technology has been explored to aid motivation and engagement in rehabilitation [5]. Gameplay can provide enhanced biofeedback and has been used to aid motor skill acquisition in self‐feeding [6]. Exergaming describes digital games that require body movements to play the games [7]. Exergames may improve lower limb muscle strength and children with unilateral CP improved handgrip, when used in addition to usual care. There is a need for larger studies with robust methodology to establish the evidence base for exergaming [8].

**Figure 1:**
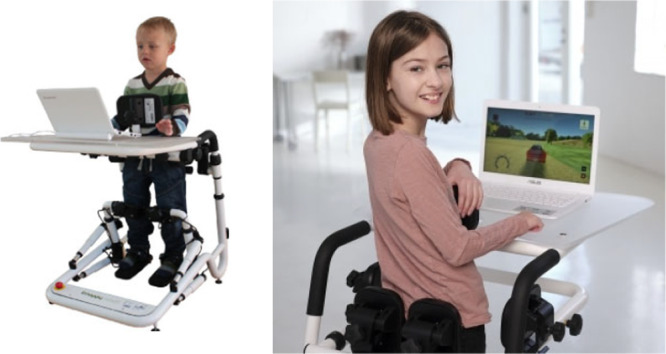
Happy Rehab™ (Innovaid, Denmark) interactive training device.

This paper reports the findings from an embedded (not a stand‐alone) qualitative study of those participating in a feasibility randomised controlled trial (fRCT) (the ACCEPT study). This qualitative work explored their experiences of participating, their views of the trial processes and the acceptability of the 10‐week physiotherapy intervention using the Happy Rehab™ (Innovaid, Denmark) exergaming device (Figure 1) [9]. The intervention aimed to improve walking and balance by training in the Happy Rehab™ by controlling a series of games using side‐to‐side weight shift, knee and ankle movement. Quantitative feasibility results are reported separately.

Understanding the lived experience of participants is essential to designing an acceptable, effective RCT [10]. Qualitatively exploring the reasons for participating or declining and the experience of participation of children, parents and physiotherapists was core to ACCEPT.

## 2 Materials and Methods

A pragmatic, qualitative approach was undertaken using e‐diaries and interviews, incorporating aspects of photo‐elicitation. The study was conducted in accordance with COREQ guidelines [11] and was approved by the North of Scotland Research Ethics Committee (20/NS/0018).

**Figure 2:**
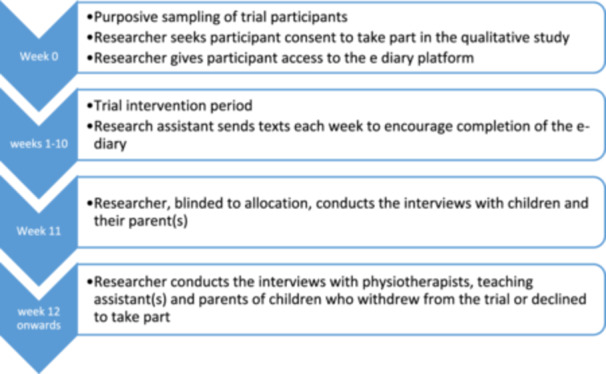
The qualitative study flow diagram showing time points nested within the feasibility RCT.

Data generation began with e‐diaries submitted weekly between weeks 1‐10 in addition to semi‐structured interviews incorporating photo‐elicitation [12], at week 11 (Figure 2). Decliner and withdrawer interviews were undertaken where possible. Field notes recorded thoughts and observations made during the process of data collection.

The lead researcher (RR) is a female paediatric physiotherapist, trained in depth interviewing. She was known to some child participants through physiotherapy clinics and assessment during the feasibility trial. All participants were aware she was undertaking her PhD.

### 2.1 Sampling and Recruitment

Purposive sampling via a sampling matrix acquired a representative view of the control and intervention groups and of different levels of GMFCS [3].

Ten parent–child dyads were recruited by RR (30% of the total feasibility study sample): both parents were eligible to participate. Three physiotherapists, who delivered the intervention, were purposively sampled. Two parents who declined or withdrew their child from the study were invited to participate in interviews.

During their first face‐to‐face fRCT appointment, child‐parent dyads were invited to participate in the qualitative component. Informed consent or assent was sought from parents (participating, declining or withdrawing) and physiotherapists and assent from children. RR undertook a qualitative assessment after the 10‐week intervention period (Figure 2).

### 2.2 E‐Diary and Photographs

With help from their caregivers, children could record semi‐structured e‐diary entries based on the prompts ‘How do you feel today?’ and ‘How was your training today?’. They could also take photographs representing their experiences of their exercise programme. The e‐diary format and questions were co‐designed by the Patient Public Involvement Advisory Group (PPI‐AG). Parents could also enter comments in the diary, marked separately by their own cypher. Weekly reminders were sent after each exercise session to complete the diary. Diary entries and photographs were uploaded directly online and managed by the clinical trials unit. Paper versions were available for return by post. An anonymous trial code linked diary and interview data.

### 2.3 Interviews

Interview topic guides were developed and informed by the literature and PPI‐AG. Interviews were conducted with the child before interviewing their parents. The children's interviews commenced by inviting them to talk about their photographs (if any), why they had chosen them and how they felt about them; then open‐ended questions were asked using the topic guides. If parents/carers answered for the child, the interviewer refocused attention back to the child and sought their response. Children using non‐verbal communication aids were interviewed with their facilitator (e.g. teaching assistant, parent); a set of questions were shared before the interview to allow preparation of speech. Parent interviews explored their experiences of participating in the trial and the effect on family life of undertaking the intervention or home exercise programme.

Participants who declined to take part or withdrew from the trial were sensitively approached to ask if they would consent to a 5‐min phone call to help identify and remove barriers to participation and explore how to make participation more accessible and attractive.

All interviews were audio‐recorded and conducted by RR in the participants' chosen environment, either face‐to‐face or by video call; interviews lasted between 20 and 45 min.

### 2.4 Data Analysis

Anonymised transcripts of interviews, photographs, diary entries and field notes were transcribed, stored on encrypted hard drives and imported into a qualitative data analysis computer software package, NVivo™, to enable the organisation and analysis of the data. Qualitative data (diary responses, photographs and interview data) were analysed in three separate groups: child, parent and physiotherapist. Textual data (diary and interviews) were analysed using reflexive thematic analysis methods [13]. Photographs were analysed descriptively (e.g. setting, description of the activity and appearance of the child).

The first analysis step involved familiarisation with the text, and then RR coded the text by allocating the text fragments to codes. Codes were revised during the process of reading the transcripts and ideas for themes were developed. For rigour, a sample of data was analysed by a second researcher (BC, female, academic children's nurse). After this, codes were reviewed, and preliminary themes formulated. These preliminary themes were shared with individual participants so they could judge whether interpretations reflected their experiences. Based on their feedback and the supervisors acting as a ‘reflecting team’, the preliminary themes and sub‐themes were condensed and revised resulting in five overarching themes with 13 sub‐themes.

Children were assigned pseudonyms which are used throughout the findings to ensure anonymity.

## 3 Results

Nine parent–child dyads were recruited: five from the intervention group (*n* = 3 urban, *n* = 2 rural) and four from the control group (*n* = 3 urban, *n* = 1 rural). Four children had coexisting additional needs. One parent consented to be interviewed but declined consent for his son to participate (Table 1). One family who consented withdrew after baseline assessment, due to their child becoming ineligible for the trial. One parent–child dyad who consented to be interviewed dropped out due to personal circumstances. One repeat interview was carried out with Bella, to enable recall of her experiences. Three physiotherapists who delivered the intervention and control arms of the RCT were interviewed (Table 2). Two interviews were conducted in a clinical setting, one in the family home and the rest took place over secure video calls with no other people present.

**Table 1:** Demographics of parent and child participants.

Child's pseudonym	Age (years)	Group	Impairments affecting the child's communication	Parent	Location
Alfie	10	Intervention	No impairment	Mother	Urban
Bella	16	Intervention	Hearing impairment	Mother	Urban
			Learning disability		
			Makaton		
Caleb	7	Intervention	No impairment	Mother	Urban
Daisy	14	Intervention	No impairment	Father	Rural
Ethan	13	Intervention	Learning disability	Mother	Rural
Freddie	7	Control	Autistic spectrum disorder	Father	Urban
Gabby	7	Control	No impairment	Mother	Rural
Harry	9	Control	Learning disability	Mother	Urban
			Autistic spectrum disorder		
Isaac	11	Control	No impairment	Father	Urban
Joseph	12	Declined to participate	Not recorded	Father	Urban

**Table 2:** Demographics of physiotherapist participants.

Physiotherapist code	Physiotherapy experience (years)	Paediatric experience (years)	Location of work
Physio 1	20	18	Community
Physio 2	9	6	Special School
Physio 3	15	6	Community

### 3.1 Photographs

Four participants uploaded photographs to their diaries; Gabby and Freddie had pictures of themselves representing their usual care and Alfie and Caleb using the Happy Rehab™ at home. Gabby looked happy in her photographs exercising against the wall at home and climbing a large wooden obstacle in an outdoor setting. Freddie was in a classroom surrounded by toys, looking like he was concentrating on trying to kick a toy with his foot. Caleb's photograph showed him in his kitchen where he had a smile on his face, apparently concentrating on the game he was playing using the Happy Rehab™. Alfie's photograph showed him in the Happy Rehab™ in his lounge, with an un‐smiling look of concentration on his face. Their expressions reflect the views they expressed during their interviews. Caleb enjoyed the games and found them motivating, whereas Alfie only liked one game and became frustrated when the device did not work properly.

### 3.2 Themes

**Figure 3:**
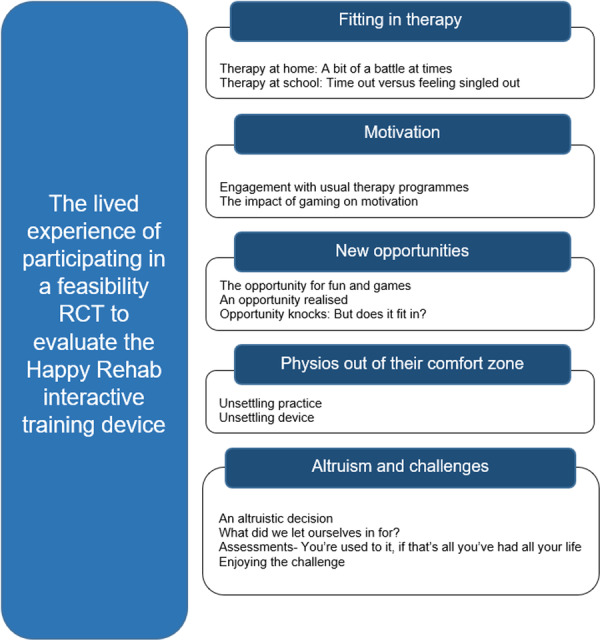
Themes and subthemes arising from the interviews.

Five main themes and their subthemes (Figure 3) draw together the findings from children, parents and physiotherapists: ‘Fitting in therapy’; ‘Motivation’; ‘New opportunities’; ‘Physios out of their comfort zone’; and ‘Altruism and challenges’.

#### 3.2.1 Fitting in Therapy

This theme explores the tension between being the child's parent and the expectations and requirements associated with delivering the child's physiotherapy at home. Parents talked about the important role that school has in supporting their child's therapy but noted that it was sometimes at the expense of missing time in class.

##### 3.2.1.1 Therapy at Home: A Bit of a Battle at Times

All but one of the parents agreed that it was difficult to find time to fit in therapy at home and it was difficult to motivate their children to engage with their exercises, as Isaac's father explained:

Doing the exercises can be a bit of a battle at times… ‘come on, just take 10 minutes out of that [games consul] to do your exercises’.

(Isaac's father)

Harry's mother said short exercises fitted into care routines, are acceptable to her son. Trying to persuade children to do therapy sometimes created conflict in the parent–child relationship. Gabby's mother described how Gabby (aged 7 years) preferred doing therapy at school as she enjoys missing class, but strongly resists doing the same exercises with her mother:

It's the opposite of at school ‐ you're getting out doing something boring… Whereas at home, it's like… ‘why do I have to do this?’ and ‘I hate you mummy’.

(Gabby's mother)

##### 3.2.1.2 Therapy at School: Time Out Versus Feeling Singled Out

Parents of children attending primary or special schools described how the therapy programme is carried out at school with the support of the teaching assistant (TA). Gabby talked with excitement about missing class and having fun therapy sessions with her TA:

I feel a bit pleased because I miss a bit of my learning!.

(Gabby, 7 years)

Isaac also talked about the liking to miss lessons. However, most mothers and fathers wanted to minimise the impact of their children missing lessons. Daisy's father was concerned about Daisy (aged 14) having to do things differently from peers, such as missing lessons and that using the Happy Rehab™ in school is a visible marker of Daisy's disability making her vulnerable to bullying:

…the trouble is it singles her out then… and she gets enough stick from time‐to‐time at school because of obviously the disability, so we didn't really want to emphasise that anymore.

(Daisy's father)

So, for Daisy and her father, Happy Rehab™ would be acceptable at home but not school.

#### 3.2.2 Motivation

All participants discussed intrinsic and extrinsic rewards, achieving their goals and the impact that gaming has on motivation and engagement.

##### 3.2.2.1 Engagement With Usual Therapy Programmes

Parents spoke of the difficulty motivating their children to do their physiotherapy programmes at home, especially where they found them boring and repetitive. Gabby and Ethan's mothers spoke about the need to use bribes and rewards to motivate their children. Several children talked about the repetitive nature of their physiotherapy exercises; for Ethan this meant that it was ‘boring’, and for Isaac it was painful at times:

Some moves kind of hurt me… it's my physio and I have to do it.

(Isaac, aged 11)

Freddie's father described how the familiarity and predictability of doing the same exercises was very comforting for Freddie, because Freddie has autism. Freddie and Bella explained that they find it fun to play while exercising.

##### 3.2.2.2 The Impact of Gaming on Motivation

Parents, children and physiotherapists unanimously supported gaming to motivate children to engage in physiotherapy. Ethan's mother thought that the distraction of the game would help. Isaac and Daisy agreed that they were likely to do more exercise if there were computer games integrated into the exercise. Caleb's mother explained the impact of gaming using the Happy Rehab™ on Caleb:

…he's actually enjoyed it, and he's actually wanted to do it every day.

(Caleb's mother)

Ethan found the competitive nature of gaming compelling:

…you got to get a certain amount of stars each time… it's quite competitive…you tried to beat your record of stars each time.

(Ethan, aged 13)

This compulsive element of gaming described by Ethan seems to represent more than just distraction or entertainment.

#### 3.2.3 New Opportunities

This theme describes the thoughts and feelings of the parents and children who took part in the intervention group, and the physiotherapists who delivered the novel intervention.

##### 3.2.3.1 The Opportunity for Fun and Games

All the physiotherapists said the Happy Rehab™ offered something different and appealing that might get children exercising more. Physio 2 identified the ‘instant feedback, in a visual way’ from the screen as an important concept that may help stimulate movement. Physio 1 talked positively about the potential benefits of the Happy Rehab™ for practicing weight transfer skills in standing:

… that's what's hard to do to simulate in hands‐on therapy…

(Physio 1)

Bella, Ethan, Caleb and Alfie all enjoyed some of the games. However, Alfie found some of the games too hard and he ultimately found this demotivating:

I did [enjoy it] at first, but … the games were really hard. Space invaders was good and that's it!.

(Alfie, aged 10)

According to his mother, a factor influencing Alfie's opinion of the Happy Rehab™ was that he disliked feeling restricted in the device and had hurt himself trying to get out unaided. He sustained bruises (reported as an adverse event); these resolved within a week.

##### 3.2.3.2 An Opportunity Realised

Many of the children found that playing the games with their leg movement intuitive. However, Bella needed some additional support to learn and understand that she was controlling the game with her movement. Her mother explained:

She wasn't aware that she was controlling until she did it a few times … and then she got it!.

(Bella's mother)

Ethan, Caleb and Bella's mothers recognised that the device helped to increase their children's ankle range of movement. Caleb's mother talked about how her son responded to the new device:

…I do think he has benefited from it… I thought his flexibilities were a bit better…he wasn't so tight.

(Caleb's mother)

Physio 2 noticed that the games helped Bella to shift her weight more evenly over each foot. Two physiotherapists queried whether all the activity was good, for example, Physio 3 was concerned about the level of selective motor control when children are playing the games:

… it feels like quite a lot of generalised movement… in terms of isolated muscle control and movement, it's really quite difficult to [know] if it's being that specific.

(Physio 3)

Three of the four children who trained on the Happy Rehab™ (Ethan, Alfie and Bella) experienced some issues with either apparent loss of power, or difficulty controlling the game, e.g. due to a faulty footplate sensor:

We couldn't always get the ship down to refill with bullets.

(Ethan's mother)

The loss of power experienced by Alfie and Bella were related to incorrect calibration of the device. This was identified early in the trial as the issue causing lack of power and additional training was put in place for physiotherapists delivering the trial.

##### 3.2.3.3 Opportunity Knocks: But Does It Fit In?

All parents and physiotherapists agreed that despite the Happy Rehab™ being large, they were willing to accommodate it for 10 weeks. Alfie's mother represented the view of all the parents:

It's OK if you got space for it, like it was out of the way for me. It was in the corner. So, it didn't really bother us…

(Alfie's mother)

Physio 2 talked about a child who had difficulty accessing the Happy Rehab™ as it was on a different floor of the house. The insurmountable block for Daisy was that they could not get the device into the house:

…it was the internal front door that's too narrow. Yep, we couldn't then get any further into the house from there, so it's a real shame.

(Daisy's father)

Physio 1 suggested that it could be set up at the clinic base for families to use. However, this was not practical for Daisy to attend the clinic three times per week. Daisy's father and Ethan's mother agreed that the additional travel time was not feasible for their families. The parent whose child withdrew declined to have the Happy Rehab™ in December, as there was not enough room for the device and a Christmas tree.

#### 3.2.4 Physios out of Their Comfort Zone

This theme explores the experiences of the physiotherapists developing confidence using the new device and feeling unsettled when things went wrong. During the fRCT, the three local NHS physiotherapists trained to use and adjust the Happy Rehab™. They all agreed that the initial training met their needs; however, putting the device into practice was more problematic.

##### 3.2.4.1 Unsettling Practice

Physio 2 describes her experience with the Happy Rehab™, and how unsettling it was not being able to feel which movements the child was making:

I find that [setting the exercises resistance] quite difficult. I think that was probably one of the hardest things that we had to do, because I couldn't feel it myself.

(Physio 2)

All three physiotherapists found some difficulty with problem‐solving when things went wrong. Under usual circumstances, they would have contacted the product representative to help solve these problems. However, the combined effects of Britain's exit from the European Union and the COVID‐19 pandemic meant that support from the UK distributor was not available.

##### 3.2.4.2 Unsettling Device

All three physios experienced times when the device stopped or when the child struggled to operate the games. Physio 1 mentioned that this might affect the trusted relationship with the parents. Physio 2 considers the impact this would have if it happened during a treatment session:

If you are setting it up … then it suddenly doesn't work, do you cancel that session and then try and find someone to sort it there and then? … You don't want to waste that appointment.

(Physio 2)

#### 3.2.5 Altruism and Challenges

This theme encompasses the selfless act of deciding to do something for the greater good and living with the consequences of that decision. This included the result of randomisation, the commitment to keep a diary and the challenge of the assessments for the children participating.

##### 3.2.5.1 An Altruistic Decision

Three parents expressed the altruistic desire to contribute to make things better for other families and children in the future. Daisy presented a pioneering attitude about testing the new device on behalf of her peers. All participants spoke with understanding of and acceptance of the need for a control group and randomisation to decide group allocation:

Even if they were just in the control group, it was still nice to know that this study was going on…you can't do research without the control, can you?.

(Gabby's mother)

##### 3.2.5.2 What Did We Let Ourselves in For?

There were several instances where the consequences of the decision to participate in the trial led to disappointment or frustration. Gabby was disappointed not to have the ‘robot physio’ and Harry's mother echoed this:

…we were a little bit disappointed that we didn't get the other group. But it is what it is, and it helps you with your study…

(Harry's Mother)

Although Freddie's father found the online diary simple to use, this view was not shared by Caleb, Alfie and Gabby's mothers. Alfie's mother suggested a weekly progress summary would be sufficient, while Gabby's mother proposed that a handheld diary would be easier to use.

##### 3.2.5.3 Assessments – ‘You're Used to Them, If That's All You've Had All Your Life’

The fRCT included four tests of balance and clinical impairment. The Next Step test involved stepping to a target. Bella's mother described how Bella took a while to understand the test and held herself unnaturally until she felt comfortable with the test. Alfie disliked how The Next Step tested his balance to the limit, explaining ‘*… it felt like you were about to fall over’*.

Harry, Gabby and Freddie disliked how the gait analysis equipment felt. Freddie and Harry did not like having markers attached to their bodies:

I like it without them [coda markers] … I don't like how it feels.

(Freddie, aged 7)

Gabby was concerned about the sticky tape and how it felt to have it removed from her skin. Freddie and Harry's parents both said that their children would have benefitted from more detailed information about the tests, so that they could prepare their children for each stage. Although children were invited to tell the physiotherapist in the assessment session to stop if any move became uncomfortable, this rarely happened. However, during the interviews, Freddie expressed his dislike of the fast stretches associated with the range of motion measures and Gabby and Isaac talked about aspects of the tests that hurt them. There was a sense of passive acceptance towards tolerating these uncomfortable parts of the assessments. Caleb's mother encapsulated this situation by saying:

He's used to it, he doesn't like people pushing his legs around, but if that's all you've had all your life…

(Caleb's mother)

##### 3.2.5.4 Enjoying the Challenge

Physio 3 was concerned about the 90‐min assessments. She thought that they might be too demanding for the children. Daisy and Bella's parents considered the length of the assessment acceptable. Gabby, Freddie, Caleb and Harry's parents agreed that their children coped well despite the assessment being 90 min long and Isaac noted:

A bit time consuming but it was quite fun.

(Isaac, aged 11)

Caleb's mother thought the sessions were tiring for her son. Harry's mother enjoyed seeing him undertaking gait analysis but stated that Harry would never be able to do all the tests, because of his autism. Caleb, Ethan and Daisy enjoyed the Next Step, where a noise sounded before a target randomly lit up that they then stepped onto. Ethan remarked:

It's quite fun. I liked that. You didn't know what number was going to go off then…It might have helped my reaction time.

(Ethan, aged 13)

Apart from Harry and Freddie, the children enjoyed most aspects of the assessments. Gabby's mother commented that the encouragement that Gabby received during the assessments was received as positive validation of her power and skills, and that this contrasted to the backdrop of her child's disability being perceived as weakness making her somewhat ‘less than’ her peers.

The parent who was interviewed after he declined for his child to participate was very supportive of the Happy Rehab™ in principle. He expressed support for gaming and felt that this would be of huge benefit to his child. He explained that the reason he declined was because of the trial design:

I think the only concern we had was that it would be in place of his usual physio. And we've seen in the past, if we don't do daily physio with Joseph, he really suffers from that… his legs will be crouching a lot more and he won't be able to stretch them out properly.

(Joseph's father)

This father indicated that then he would have consented to his child participating if the trial was designed with the new intervention in addition to his child's usual care.

## 4 Discussion

Children with CP typically undertake physiotherapy programmes aimed to improve or maintain their motor skills. These programmes require time and effort by the child and their adult supporters at school and home. This study used qualitative approaches to explore the feasibility of an RCT to improve balance and walking. This is recognised as a valuable way of exploring uncertainties and optimising the intervention in preparation for a full trial [10]; however, it is relatively uncommon in fRCTs for children with CP [14, 15]. This study sought the opinions of children on the intervention and trial procedures, which will help to make the RCT more accessible and enjoyable for the participants.

Most children were highly motivated by the badges and virtual rewards in the Happy Rehab™ games. Parents and children saw gaming as a usual part of children's life that should be incorporated into physiotherapy. There is an emerging body of evidence to support ‘serious games’ in therapy [6, 16]. Studies have shown that the inclusion of gaming such as Virtual Reality games can increase exercise repetitions and help children to track their achievements [6, 17]. However, one child was not motivated by the games because he found them too hard and he experienced faults with the device. The insights from the children highlight the need for the physiotherapist to tailor the training at the right level of difficulty, called the ‘just right challenge’ [18]. Where the challenge is too high or perceived as unachievable, this can have a demotivating effect.

Participants showed support for the Happy Rehab™ as a tool for motivating children to exercise. This is important as factors such as motivation and autonomy impact on the ability of a child to adhere to exercise programmes [19]. The intervention also reduced the burden on parents carrying out manual therapy with their children. Previous work reveals parents feeling unwelcome pressure to support their children to carry out home exercise [20].

Nearly all parents were willing to accommodate the device for a 10‐week period, except when it was in direct competition with the family's needs for space, such as at Christmas. Parents have previously expressed a willingness to sacrifice space or the aesthetic of their home to accommodate large equipment, where they consider it benefits their child [21]. Some parents felt that the Happy Rehab™ should not be school‐based as it might single their child out [22, 23]. This reinforced the need to have the option to train at home, school or clinic in the full RCT.

A key component of the feasibility of the intervention was the clinician's ability to set up the intervention and coach carers to use it. Physiotherapists expressed their lack of experience with the Happy Rehab™ meant that they did not fully establish the confidence to set up and tailor the programme for individuals at home. Previous studies have shown the importance of pre‐trial training and education for clinicians using manualised interventions [24]. Innovation champions may facilitate clinicians in adopting novel rehabilitation technology [25]. Therefore, a future trial would benefit from a more detailed and flexible training programme for physiotherapists, as well as a Happy Rehab™ ‘champion’ with in‐depth skills to support their colleagues.

Recruitment to the fRCT was reduced due to the COVID‐19 pandemic and caused some limitations to this embedded study. This smaller sample size limited the potential qualitative dataset, meaning that saturation may not have been reached. The results may not be representative of the diversity of this population and are not therefore generalisable. The interviewer used photo elicitation to help the children to be more expansive about their experiences. The relatively low numbers of photographs uploaded via the diaries may have limited the data obtained from participants.

The interviewer's status as PhD candidate and clinician may have introduced bias and potential power imbalance with interviewees. The interviewer was conscious of this during interviews and encouraged participants to express both positive and negative aspects of their experiences. The interviewer had prior knowledge of the children's therapeutic exercise, which may have biased data collection or interpretation. A further limitation was that transcripts were not returned to participants for validation. Recommendations for future studies would be to increase the sample size, use an independent interviewer and for participants to validate the interview transcripts.

This study interviewed children to help create ethical research that is acceptable to them and to develop effective interventions [26]. Home exercise programmes constitute 50%–80% of therapy; however, adherence to home programmes is often poor [27]. Child participants talked about complying with home exercises that sometimes hurt them and how gaming might be a more motivating to exercise. Most children found the assessments acceptable and an enjoyable challenge. However, some autistic participants struggled to complete assessments that included body markers using adhesive tape [28]. This finding has not been previously reported in the literature. Recommendations for the full trial include shorter neurodivergent‐friendly assessments, omitting body‐marker movement analysis and the inclusion of suitable pre‐test explanations to prepare children for assessments. Their experiences with the device and trial processes will shape the future trial with children as partners in developing appropriate, ethical

A strength of this study was the inclusion of children with additional communication needs by engaging with their adult communication partners. Bella's mother supported her to use Makaton, and Harry's mother helped interpret his reactions to memories of the trial. This approach allowed the study to be more inclusive of participants, including those with learning disability and autism, who could not have been interviewed without support. Although these children tended to give quite concrete and short responses, they were very clear when indicating their like or dislike of certain procedures. This was invaluable when considering the feasibility of a trial in children with CP.

## 5 Conclusion

Overall, the intervention was acceptable to children and parents. Parents were mostly willing to accommodate the device for 10 weeks. Children found the gaming aspect both motivating and enjoyable but some children required shorter neurodivergent‐friendly assessments. Physiotherapists require more support to solve technical problems. A full evaluation of the Happy Rehab™ requires additional technical support and experienced ‘champion’ users of the device.

## Author Contributions

**Rachel Rapson:** conceptualisation, investigation, funding acquisition, writing – original draft, methodology, validation, visualisation, writing – review and editing, formal analysis, project administration, data curation. **Jos M. Latour:** conceptualisation, funding acquisition, methodology, validation, visualisation, writing – review and editing, supervision. **Jonathan Marsden:** conceptualisation, funding acquisition, methodology, validation, writing – review and editing, supervision, project administration. **Bernie Carter:** conceptualisation, funding acquisition, writing – original draft, methodology, validation, visualisation, writing – review and editing, formal analysis, supervision.

## Ethics Statement

This study was approved by the North of Scotland Research Ethics Committee (20/NS/0018). Innovaid (Denmark) has given written permission for the publication of their photographs.

## Conflicts of Interest

The authors declare no conflicts of interest.

## Data Availability

The data that support the findings of this study are openly available in PEARL at https://pearl.plymouth.ac.uk/hp-theses/10/.
